# WhySchool project: effects of a school-based mental health literacy programme on teachers and school health professionals

**DOI:** 10.1192/bjo.2026.12009

**Published:** 2026-07-06

**Authors:** Joana Moreira, Virgínia Conceição, Lars Mehlum, Stanley Kutcher, Ricardo Gusmão

**Affiliations:** EPIUnit ITR, Institute of Public Health, University of Porto, Portugal; National Centre for Suicide Research and Prevention, Institute of Clinical Medicine, University of Oslo, Norway; Department of Psychiatry, Faculty of Medicine, Dalhousie University, Canada; Department of Public Health and Forensic Sciences, and Medical Education, Faculty of Medicine, University of Porto, Portugal

**Keywords:** School mental health, mental health literacy, teachers, school health professionals

## Abstract

**Background:**

Adolescent mental health is a growing public health concern due to the high prevalence of mental disorders, many of which remain unrecognised and untreated. School staff are strategically positioned to promote mental health, recognise mental health problems and support pathways into care, but often lack sufficient mental health literacy (MHL) and confidence to act.

**Aims:**

This study evaluated the effects of the WhySchool project, a school-based programme to promote MHL among teachers and school health professionals (SHPs).

**Method:**

We implemented WhySchool in 72 public middle and high schools across Portugal through a cascade training approach. With a pre–post design, we assessed 788 teachers and 201 SHPs on mental health knowledge (MHK), personal depression stigma, openness to seeking help and confidence in identifying/referring students. Paired-sample *t*-tests with Cohen’s *d* estimated changes, and generalised linear mixed models (GLMMs) accounted for confounders and within-subject variability.

**Results:**

The programme was associated with significant improvements in all outcomes across both professional groups, with moderate-to-large effect sizes (MHK *d* = 1.12 (95% CI 1.04 to 1.20); stigma *d* = −1.05 (95% CI −1.12 to −0.97); openness *d* = 0.44 (95% CI 0.37 to 0.51); confidence *d* = 0.87 (95%CI 0.79 to 0.94)). GLMMs confirmed these results. Gains varied across professional groups and demographic characteristics, with those having lower baseline scores generally benefiting most.

**Conclusions:**

The WhySchool resulted in observable improvements in teachers’ and SHPs’ MHL, including increased knowledge, reduced stigma, improved help-seeking attitudes and strengthened confidence to support students. The cascade model provides a viable and sustainable strategy for large-scale implementation, empowering educational communities to better support student mental health.

Adolescent mental health is a critical and growing public health concern. Worldwide, nearly 19% of young people aged 15 to 19 suffer from a mental disorder,^1^ yet these conditions often go unrecognised and untreated.^2^ Globally, mental disorders are the leading cause of years lived with disability,^1^ and suicide tragically stands as the second cause of death among this age group.^1^ As most mental disorders emerge before the age of 25, early recognition and timely intervention are paramount to altering their trajectories and mitigating long-term adverse consequences.^3,4^

Adolescents spend most of their time at school, lending teachers and school health professionals (SHPs) the unique position to act as first-line gatekeepers.^5–9^ The education system is strategically positioned to promote positive mental health, recognise mental health problems and support pathways into appropriate mental health care.^5,7^ Yet, young people face numerous barriers to help-seeking, including difficulties in identifying symptoms and pervasive stigma.^4,10^ As teachers and SHPs are often the first to notice these difficulties, enhancing their capacity to respond effectively has become essential.

To address this, mental health literacy (MHL) has emerged as a key competency for school staff.^11,12^ MHL is a multidimensional construct encompassing four core components: understanding how to obtain and maintain positive mental health; recognising mental disorders and their treatments; reducing the stigma associated with mental health issues; and improving help-seeking behaviours.^5^ Enhancing MHL among school staff offers significant dual benefits: first, professionals with higher MHL are better equipped to recognise students’ mental health challenges, guide them to timely and appropriate care and act with greater confidence;^6,13,14^ second, increased MHL also empowers them to understand and manage their own mental health problems and seek timely interventions, fostering a more resilient and competent school environment overall.^15–17^

However, research shows that teachers lack adequate training and are not well prepared for such a role.^18–22^ Limited mental health knowledge (MHK), low confidence in supporting students potentially in need of help and holding stigmatising attitudes towards mental illness are common among teachers.^23–26^ Evidence also suggests that MHL levels may be influenced by demographic and experiential factors, such as gender, years of professional experience and prior mental health training.^14,27,28^ Understanding how these characteristics affect training outcomes is vital for designing interventions that are both effective and equitable.

In response to these needs, numerous school-based MHL interventions have been implemented globally, particularly targeting teachers. While many studies show promising results, both the diversity of intervention designs and the methodological limitations of existing evaluations constrain comparability and generalisability, underscoring the need for more rigorous and harmonised evidence to guide scalable and sustainable implementation.^6,14,29^ Critically, many programmes focus primarily on improving knowledge and attitudes towards mental illness while neglecting a crucial barrier to effective action: teachers’ confidence in identifying and supporting students with mental health needs, thus impairing the effectiveness of the intervention by potentially leaving vulnerable students without the necessary support.^29^ Moreover, teachers’ personal perspectives regarding help-seeking influence their willingness and intention towards referring students, with more positive help-seeking attitudes serving as an important precursor to proactive professional gatekeeping, underscoring the need for targeted school mental health training that addresses these factors.^30^

Building on these challenges, the current study addresses a well-recognised implementation gap in translating school-based MHL programmes into consistent, scalable and sustainable practice. Specifically, we present results from a comprehensive school-based MHL programme for teachers and SHPs developed within the scope of the Why Youth Mental Health Care School-Based with Primary Care Liaison (WhySchool) project,^31,32^ which was developed in collaboration with and culturally adapted from previous work that led to the ‘Mental Health and High School Curriculum Guide’.^12,33^ Our intervention targeted all four core MHL components, with particular attention to building professional confidence alongside knowledge and attitudes. We aimed to (a) evaluate improvements in mental health knowledge, personal stigma towards depression, openness to seeking help and confidence in identifying and referring students in need of support; (b) assess how these effects varied between professional groups and training levels, comparing teachers with SHPs and recipients of different training levels in the cascade model; (c) explore the influence of baseline participant characteristics on training outcomes; and (d) evaluate participant satisfaction with the programme.

## Method

### Study design and participants

We used a pre–post evaluation design with the post-test conducted one to two weeks after the training concluded to assess immediate knowledge retention and changes in attitudes and confidence. We recruited teachers and SHPs available to participate from 72 public middle and high schools across mainland Portugal using a convenience sampling strategy to optimise logistical feasibility and school participation availability.

The training programme was implemented in two phases: (a) WhySchool I (WS I, 2015–2016), delivered across six regions of mainland Portugal (North, Centre, Alentejo, Lisbon, Setúbal Península and the Algarve), and (b) WhySchool II (WSII, 2018–2020), conducted in 11 municipalities within the Porto metropolitan area (northern Portugal). Both phases used identical training content and procedures.

### Intervention

WhySchool^31,32^ is a mental health promotion and mental illness prevention programme for professionals in Portuguese public middle and high schools. The intervention aims to increase school-based MHL by building capacity in teachers and SHPs to act as strategic gatekeepers. This approach focuses on their professional role in supporting students while simultaneously enhancing their own literacy as a necessary foundation for an integrated and sustainable school-wide approach to mental health.

The intervention curriculum is structured into twelve modules that address key areas aligned with the four core MHL components, including stress management, adolescent mental health, stigma reduction and help-seeking behaviour (Supplementary Table S1). In-person sessions of 4 h provide the training framework, combining a structured didactic foundation with active, collaborative methods.

To enhance sustainability and expand the training’s reach, we employed a ‘train-the-trainer’ cascade model for teachers. Experienced MHPs initially trained school heads, coordinators and master’s-level teachers for 16 h (Level 1). These Level 1 participants subsequently delivered 8 h of training to their teaching colleagues (Level 2), thereby building internal capacity within school communities. SHPs received 8 h of training, delivered directly by MHPs, without utilising the cascade model.

### Instruments

We evaluated the effects using the following self-administered questionnaires.

- The Mental Health Knowledge (MHK) questionnaire, a 31-item true/false measure to evaluate general knowledge about mental health, mental disorders and treatments. The research team translated and culturally adapted the Canadian resource ‘Mental Health and High School Curriculum Guide’^12,33^ to ensure alignment with the Portuguese context and the WhySchool programme (Cronbach’s α = 0.89) while maintaining the original measure’s core content, with the total score reflecting the number of correct answers. Total scores were converted to percentages, with higher scores indicating greater knowledge. More information about the psychometric properties of the Portuguese version can be found in the supplementary file (Tables S2–S4).
- The Portuguese version of the Personal Depression Stigma Subscale (pers DSS)^34^ to assess one’s beliefs and attitudes towards depression. This subscale comprises 9 items rated on a 5-point Likert scale, ranging from ‘strongly disagree’ to ‘strongly agree’. Following the original scale guidelines and the Portuguese validation, total scores were converted into percentages, with higher scores indicating greater personal stigma.
- The Openness to Seeking Help questionnaire assesses participants’ willingness to seek help for psychological or emotional problems for themselves. To capture this construct, we employed brief Likert-type items evaluating agreement with statements such as ‘I would be likely to seek help if I were concerned about my mental health.’ In the WSII, 2018–2020, this measure was expanded to include a 5-item subscale from the Attitudes Toward Seeking Professional Psychological Help (ATSPPH) questionnaire,^35,36^ allowing for a more nuanced assessment. To ensure comparability across items and study phases, all scores were standardised to percentages, with higher values indicating greater openness.
- The Confidence scale in identifying and referring students who are potentially in need of help. Based on the Morriss Confidence Scale,^37^ the scale consists of items rated on a 10-point Likert scale from ‘not at all confident’ to ‘very confident’, resulting in a score range from 2 to 20.

We also collected information regarding participant characteristics, including profession, age, gender, school regional area, teaching area, grade level taught and previous mental health training.

Last, we assessed training satisfaction using an eight-item questionnaire, rated on a six-point Likert scale from ‘not at all adequate’ to ‘completely adequate’, covering logistics, trainer quality and content relevance. We also included an overall satisfaction item, using a five-point Likert scale ranging from ‘not helpful at all’ to ‘extremely helpful’.

### Statistical analysis

First, we analysed participants’ characteristics and training satisfaction using descriptive statistics (chi-square for categorical variables, *t*-tests and ANOVA for continuous variables). We evaluated the programme’s effectiveness using paired-sample *t*-tests and Cohen’s d to assess pre–post changes for each outcome measure in each professional group (teachers Level 1, teachers Level 2 and SHPs). We then applied GLMMs to account for repeated measures and within-subject variability, while controlling for potential confounders such as gender, age and prior mental health training. We conducted separate analyses for each professional group, adjusting for the WhySchool implementation period and the school regional area. We performed all statistical analyses using IBM SPSS Statistics version 30.0.0 for macOS (IBM Corp., Armonk, NY, USA; https://www.ibm.com/products/spss-statistics), applying a 95% CI for statistical significance. Additional analyses examining baseline predictors, detailed demographic comparisons and exploratory correlational analyses are reported in the supplementary file.

### Ethical approval

The authors assert that all procedures contributing to this work comply with the ethical standards of the relevant national and institutional committees on human experimentation and with the Helsinki Declaration of 1975, as revised in 2013. All procedures involving human participants were approved by the Research Ethics Board of the Lisbon and Tejo Valley Health Regional Administration (ID reference 18025/CES/2015). All participants digitally signed informed consent following the Helsinki and Oviedo Conventions.

## Results

The following results focus on the core research questions regarding WhySchool’s effectiveness and the success of the cascade training model. Supplementary analyses include: missing data (Table S5) handling procedures and diagnostics; comprehensive demographic and regional comparisons between WhySchool I and II implementation periods (Table S6); exploratory correlational analyses examining relationships between baseline measures and training effects (Table S7); and detailed baseline predictors of training outcomes using generalised linear models (Table S8).

### Participant characteristics

The study sample comprised 989 participants categorised into three groups: 195 teachers at Level 1 (TL1), 593 teachers at Level 2 (TL2) and 201 SHPs, of whom 125 were psychologists. Participants were recruited across two implementation phases: WhySchool I (2015–2016) and WhySchool II (2018–2020) (Table 1).

**Table 1:** Main participant characteristics Table 1 long description.

	Teachers Level 1 (TL1), *n* = 195	Teachers Level 2 (TL2), *n* = 593	School health professionals (SHPs), *n* = 201	Total, *n* = 989
Age, M (s.d.)	48.15 (7.49)	48.14 (7.72)	41.02 (9.06)	46.69 (8.22)
Age groups, *n* (%)^a^				
≤39 years	24 (12.3)	103 (17.4)	109 (54.2)	236 (23.9)
40–9 years	86 (44.1)	244 (41.1)	57 (28.4)	387 (39.1)
≥50 years	85 (43.6)	265 (44.7)	35 (17.4)	385 (38.9)
Gender, *n* (%)				
Women	155 (79.5)	473 (79.8)	170 (84.6)	798 (80.7)
Men	40 (20.5)	120 (20.2)	31 (15.4)	191 (19.3)
School level, *n* (%)				
Middle School (7th–9th)	117 (60.0)	368 (62.0)	–	484 (61.3)^b^
High School (10th–12th)	78 (40.0)	225 (38.0)	–	303 (38.5)^b^
Teaching area, *n* (%)				
Sciences	98 (50.3)	247 (41.7)	–	345 (43.8)^b^
Humanities/Social Sciences	96 (49.2)	343 (57.8)	–	439 (55.7)^b^
NA	1 (0.5)	3 (0.5)	–	4 (0.5)^b^
Professional category, *n* (%)				
Psychologists	–	–	125 (62.2)	125 (12.6)
Other SHPs^c^	–	–	76 (37.8)	76 (7.7)
Prior mental health training, *n* (%)				
No	138 (70.8)	476 (80.3)	35 (17.4)	649 (65.6)
Yes	57 (29.2)	117 (19.7)	166 (82.6)	340 (34.4)
Implementation phase, *n* (%)				
WhySchool I (2015–2016)	87 (44.6)	411 (69.3)	104 (51.7)	602 (60.9)
WhySchool II (2018–2020)	108 (55.4)	182 (30.7)	97 (48.3)	387 (39.1)

M, mean; *n*, absolute number; NA, not applicable.

a. Cut-off point obtained from the decision trees.

b. Percentages calculated for teachers only (*n* = 788).

c. Other school health professionals include nurses and public health physicians.

Key group differences emerged in age distribution and professional background. SHPs were significantly younger (*t* = 11.69_(987)_, <0.001) and had substantially more prior mental health training experience (χ^2^ = 259.89_(1)_, *p* < 0.001) compared with teachers. Most teachers were aged 40 years or older, worked primarily in middle schools and had limited previous mental health training (Table 1). Detailed demographic comparisons by implementation phase and region, as well as baseline outcome scores and their associations with demographic variables, are presented in Supplementary Table S6.

### WhySchool training programme effects

The WhySchool training programme was associated with substantial improvements in all outcomes across the three groups, with moderate to large effect sizes (Table 2). Teachers at both training levels achieved the largest effect size in MHK, while SHPs showed the greatest improvement in personal depression stigma reduction. Openness to seeking help had the smallest gains across all groups.

**Table 2:** Baseline and post-training analysis of MHK, pers DSS, openness and confidence by profession and training level Table 2 long description.

Outcomes	Baseline	Post-training	*t*_(df)_	*p*	Cohen’s *d*		
	M (s.d.)	M (s.d.)			Point estimate	95% CI	
MHK (%)	55.13 (16.96)	79.19 (13.12)	35.21_(988)_	<0.001	1.12	1.04	1.20
TL1	54.13 (16.13)	87.06 (9.63)	24.84_(194)_	<0.001	1.78	1.55	2.00
TL2	52.80 (14.73)	77.71 (12.81)	31.20_(592)_	<0.001	1.28	1.17	1.39
SHPs	62.96 (21.16)	76.09 (14.08)	7.37_(200)_	<0.001	0.52	0.37	0.67
pers DSS (%)	32.89 (15.66)	15.90 (9.78)	−32.92_(988)_	<0.001	−1.05	−1.12	−0.97
TL1	36.64 (14.78)	16.55 (11.33)	−16.70_(194)_	<0.001	−1.20	−1.38	−1.01
TL2	31.58 (15.00)	16.35 (9.44)	−24.23_(592)_	<0.001	−1.00	−1.09	−0.90
SHPs	33.13 (17.72)	13.94 (8.91)	−15.44_(200)_	<0.001	−1.09	−1.26	−0.91
Openness (%)	68.53 (24.69)	77.83 (20.97)	13.83_(988)_	<0.001	0.44	0.37	0.51
TL1	76.24 (18.98)	82.14 (17.55)	4.04_(194)_	<0.001	0.29	0.15	0.43
TL2	65.85 (26.38)	76.56 (22.16)	12.12_(592)_	<0.001	0.50	0.41	0.58
SHPs	68.95 (22.86)	77.40 (19.94)	5.87_(200)_	<0.001	0.41	0.27	0.56
Confidence (2–20)	10.06 (4.44)	13.28 (3.75)	27.25_(988)_	<0.001	0.87	0.79	0.94
TL1	9.33 (4.32)	14.18 (3.59)	18.37_(194)_	<0.001	1.35	1.12	1.51
TL2	9.29 (3.86)	12.36 (3.46)	21.52_(592)_	<0.001	0.88	0.79	0.98
SHPs	13.02 (3.86)	15.13 (3.86)	7.57_(200)_	<0.001	0.53	0.39	0.68

MHK, mental health knowledge; pers DSS, personal Depression Stigma Subscale; Openness, openness to seeking help; Confidence, confidence in identifying or referring students; TL1, teachers Level 1; TL2, teachers Level 2; SHPs, school health professionals; M, mean.

To assess whether post-training improvements were maintained while controlling for potential confounders identified in the GLMMs and accounting for within-subject variability, we conducted GLMMs for each outcome. Only factors with a significant main effect or a significant interaction with another fixed factor were retained (Table 3).

**Table 3:** Final generalised linear mixed models for each outcome score by profession and training level Table 3 long description.

MHK		*B*	s.e.	*t*	*p*	95% CI	
TL1	Intercept	7.54	0.11	70.03	<0.001	7.32	7.74
(*R* ^2^ = 0.725)	Time	1.89	0.09	18.96	<0.001	1.70	2.09
	Gender^a^	−0.73	0.16	−4.44	<0.001	−1.05	−0.40
	Time post*Men	0.80	0.20	3.92	<0.001	0.40	1.20
	Time post*Region_Algarve^e^	−1.59	0.36	−4.47	<0.001	−2.29	−0.89
TL2	Intercept	7.19	0.07	97.85	<0.001	7.05	7.33
(*R* ^2^ = 0.626)	Time	1.21	0.10	12.75	<0.001	1.03	1.40
	Gender^a^	−0.21	0.06	−3.52	<0.001	−0.33	−0.09
	Prior training^b^	0.28	0.09	3.11	<0.01	0.10	0.46
	Time post*Prior training	−0.35	0.12	−2.92	<0.01	−0.58	−0.11
	Time post*Region_Alentejo^e^	0.47	0.14	3.52	<0.001	0.21	0.74
	Time post*Region_Centro^e^	−0.64	0.32	−1.99	<0.05	−1.26	−0.01
	Time post*WS II	0.91	0.13	7.11	<0.001	0.66	1.16
SHPs	Intercept	7.57	0.97	7.77	<0.001	5.65	9.48
(*R* ^2^ = 0.517)	Time	0.47	0.25	1.87	<0.05	−0.02	0.96
	Age 40–9^c^	0.47	0.19	2.47	<0.05	0.10	0.84
	Prior training^b^	0.45	0.15	3.04	<0.01	0.16	0.75
	Time post*Age 40–9	−0.66	0.26	−2.58	<0.05	−1.17	−0.16
	Time post*Region_Lisbon + Setúbal^e,f^	1.10	0.43	2.56	<0.05	0.25	1.95
	Time post*WS II	0.95	0.27	3.45	<0.001	0.41	1.48
pers DSS		*B*	s.e.	*t*	*p*	95% CI	
TL1	Intercept	3.34	.0731	45.81	<0.001	3.20	3.49
(*R* ^2^ = 0.518)	Time	−0.61	.0717	−7.39	<0.001	−0.67	−0.39
	WhySchool period^d^	0.47	.0921	5.10	<0.001	0.29	0.65
	Time post*WS II	−0.34	.0965	−3.51	<0.001	−0.53	−0.15
TL2	Intercept	3.31	0.04	84.06	<0.001	3.21	3.37
(*R* ^2^ = 0.539)	Time	−0.66	0.05	−14.24	<0.001	−0.75	−0.57
	WhySchool period^d^	0.54	0.05	10.09	<0.001	0.44	0.65
	Time post*Region_Algarve^e^	0.33	0.09	3.58	<0.001	0.15	0.52
	Time post*Region_Lisbon^e^	0.35	0.11	3.04	<0.01	0.12	0.57
	Time post*Region_Alentejo^e^	0.18	0.07	2.64	<0.01	0.05	0.32
	Time post*WS II	−0.22	0.06	−3.37	<0.001	−0.34	−0.09
SHPs	Intercept	3.06	0.08	38.80	<0.001	2.91	3.22
(*R* ^2^ = 0.598)	Time	−0.48	0.07	−7.37	<0.001	−0.61	−0.35
	WhySchool period^d^	0.80	0.10	7.97	<0.001	0.60	0.99
	Gender^a^	−0.20	0.10	−2.02	<0.05	−0.39	−0.01
	Time post*WS II	−0.65	0.09	−6.93	<0.001	−0.84	−0.47

Openness		*B*	s.e.	*t*	*p*	95% CI	
TL1	Intercept	9.25	0.14	64.57	<0.001	8.97	9.53
(*R* ^2^ = 0.434)	Time	−0.02	0.12	−0.19	0.85	−0.25	0.21
	WhySchool period^d^	−0.79	0.17	−4.61	<0.001	−1.13	−0.45
	Gender^a^	−0.33	0.14	−2.27	<0.05	−0.06	−0.04
	Time post*WS II	0.66	0.16	4.06	<0.001	0.34	0.97
TL2	Intercept	7.38	0.12	61.07	<0.001	7.15	7.62
(*R* ^2^ = 0.630)	Time	1.14	0.11	10.05	<0.001	0.92	1.37
	WhySchool period^d^	1.19	0.15	7.72	<0.001	0.89	1.50
	Gender^a^	−0.62	0.15	−4.21	<0.001	−0.92	−0.33
	Region_Algarve^e^	0.74	0.23	3.26	<0.001	0.29	1.18
	Region_Lisbon^e^	1.09	0.27	4.02	<0.001	0.56	1.61
	Region_Alentejo^e^	1.61	0.16	9.80	<0.001	1.28	1.93
	Time post*Region_Algarve^e^	−0.89	0.21	−4.18	<0.001	−1.31	−0.47
	Time post*Region_Alentejo^e^	−1.08	0.15	−7.09	<0.001	−1.38	−0.78
	Time post*WS II	−0.80	0.14	−5.51	<0.001	−1.08	−0.51
	Time post*Men	0.44	0.14	3.18	<0.01	0.17	0.71
SHPs	Intercept	7.57	.17	44.15	<0.001	7.24	7.91
(*R* ^2^ = 0.565)	Time	.67	.12	5.47	<0.001	0.43	0.91
	WhySchool period^d^	1.18	.21	5.64	<0.001	0.77	1.59
	Region_Algarve^e^	1.03	.32	3.18	<0.01	0.39	1.66
	Region_Lisbon + Setúbal^e,f^	0.64	.29	2.22	<0.05	0.07	1.21
	Time post*WS II	−0.39	.17	−2.32	<0.05	−0.73	−0.06

Confidence		*B*	s.e.	*t*	*p*	95% CI	
TL1	Intercept	2.02	0.19	10.49	<0.001	1.64	2.40
(*R* ^2^ = 0.722)	Time	0.92	0.15	6.13	<0.001	0.62	1.21
	MHK	0.01	0.001	5.08	<0.001	0.01	0.01
	Age ≥50^c^	0.64	0.15	4.37	<0.001	0.35	0.92
	Age 40–9^c^	0.48	0.15	3.25	<0.01	0.19	0.76
	Time post*WS II	−0.19	0.08	−2.44	<0.05	−0.34	−0.04
	Time post*Age ≥50^c^	−0.57	0.14	−4.21	<0.001	−0.84	−0.30
	Time post*Age 40–9^c^	−0.41	0.14	−2.99	<0.01	−0.68	−0.14
TL2	Intercept	2.54	0.06	40.73	<0.001	2.42	2.66
(*R* ^2^ = 0.618)	Time	0.24	0.03	7.47	<0.001	0.18	0.31
	MHK	0.01	0.001	8.79	<0.001	0.01	0.01
	Prior training^b^	0.21	0.05	4.49	<0.001	0.12	0.31
SHPs	Intercept	3.21	0.11	29.80	<0.001	3.00	3.43
(*R* ^2^ = 0.696)	Time	0.17	0.08	2.03	<0.05	0.01	0.33
	Region_Algarve^e^	0.46	0.17	2.68	<0.01	0.12	0.79
	Region_Lisbon + Setúbal^e,f^	−0.36	0.16	−2.32	<0.05	−0.66	−0.06
	Age ≥50^c^	0.26	0.10	2.51	<0.05	0.06	0.46
	Age 40–9^c^	0.34	0.09	3.64	<0.001	0.16	0.53
	Gender^a^	−0.77	0.12	−6.58	<0.001	−0.99	−0.54
	Prior training^b^	0.42	0.09	4.55	<0.001	0.24	0.61
	Time post*WS II	0.30	0.09	3.32	<0.001	0.12	0.48
	Time post*Region_Lisbon + Setúbal^e,f^	0.47	0.14	3.25	<0.001	0.18	0.75
	Time post*Age ≥50^c^	−0.30	0.09	−3.21	<0.001	−0.49	−0.12
	Time post*Age 40–9^c^	−0.38	0.09	−4.41	<0.001	−0.55	−0.21
	Time post*Men	0.54	0.11	5.10	<0.001	0.33	0.75

MHK, mental health knowledge; pers DSS, personal Depression Stigma Subscale; Openness, openness to seeking help; Confidence, confidence in identifying or referring students; TL1, teachers Level 1; TL2, teachers Level 2; SHPs, school health professionals; WS II, WhySchool II (2018–2020); *B*, beta regression coefficients. The reference groups are indicated in footnotes a–e. All models are adjusted for WhySchool period and implementation region.

a. Women

b. No.

c. ≤39 years old.

d. WhySchool I (2015–2016).

e. North.

f. For level 1 teachers and SHP groups, we merged the Centre region with the North and Setúbal with Lisbon to ensure statistical robustness, given the small number of participants in those areas. MHK, openness and confidence with probability distribution: normal; link function: power (0.5). pers DSS with probability distribution: gamma; link function: log.

### Mental health knowledge

Participants demonstrated significant post-training improvements in mental health knowledge across all groups (Table 3). However, the magnitude of these gains varied across professions and training levels.

Teachers at Level 1 exhibited the largest improvements (Table 3), with both men and women achieving significant gains (Table 4). Men who started from lower baseline levels showed greater improvements. Level 2 teachers also demonstrated substantial knowledge gains, with participants in WhySchool II and those without prior training benefiting the most (Table 3). Nonetheless, we verified noteworthy gains in WhySchool I and in those with prior training (Table 4). Regional variations emerged, with the Alentejo region showing the strongest gains and the Centre region showing more modest improvements among Level 2 teachers.

**Table 4:** Estimated means, standard errors and paired analysis of MHK, pers DSS, openness and confidence at baseline and post-training from time interactions from GLMMs Table 4 long description.

Outcomes	Professional group	Factor	Level	Baseline		Post-training		*t*_(df)_	*p*
				Estimated mean	s.e.	Estimated mean	s.e.		
MHK (%)	TL1	Gender	Women	57.38	1.69	83.24	1.75	11.19_(379)_	<0.001
			Men	46.91	2.45	84.52	2.52	10.89_(379)_	<0.001
		Region	North + Centre	50.83	1.31	88.77	1.32	21.52_(379)_	<0.001
			Alentejo	49.81	2.43	86.41	2.54	10.99_(379)_	<0.001
			Lisbon + Setúbal^a^	47.81	4.19	89.66	4.41	7.00_(379)_	<0.001
			Algarve	60.00	4.06	71.38	4.17	1.99_(379)_	<0.05
	TL2	Prior training	No	52.06	1.20	80.03	1.28	16.55_(1169)_	<0.001
			Yes	56.22	1.54	78.88	1.58	10.55_(1169)_	<0.001
		Region	North	51.92	0.83	75.66	0.85	20.90_(1169)_	<0.001
			Centre	60.09	3.53	74.09	3.69	2.76_(1169)_	<0.01
			Alentejo	54.61	1.40	87.56	1.48	16.65_(1169)_	<0.001
			Lisbon	55.03	2.44	83.16	2.55	8.08_(1169)_	<0.001
			Setúbal	49.52	3.45	76.87	3.59	5.51_(1169)_	<0.001
			Algarve	53.82	1.96	79.80	2.05	9.35_(1169)_	<0.001
		WhySchool period	I (2015–2016)	54.40	1.09	71.91	1.12	11.85_(1169)_	<0.001
			II (2018–2019)	53.83	1.65	87.38	1.77	14.07_(1169)_	<0.001
	SHPs	Age	≤39	57.12	2.66	78.63	1.88	7.33_(387)_	<0.001
			40–9	64.42	3.18	75.25	2.57	2.81_(387)_	<0.01
			≥50	60.35	2.99	74.22	3.17	4.06_(387)_	<0.001
		Region	North + Centre	61.82	1.70	71.04	1.47	4.72_(387)_	<0.001
			Alentejo	62.03	3.19	75.90	3.12	3.72_(387)_	<0.001
			Lisbon + Setúbal^a^	52.47	3.40	79.37	3.20	6.47_(387)_	<0.001
			Algarve	66.50	3.97	77.90	4.05	2.18_(387)_	<0.05
		WhySchool period	I (2015–2016)	63.03	1.82	70.59	1.69	3.70_(387)_	<0.001
			II (2018–2019)	58.20	3.22	81.77	2.98	6.03_(387)_	<0.001
pers DSS (%)	TL1	WhySchool period	I (2015–2016)	27.93	1.80	15.13	0.98	−7.56_(383)_	<0.001
			II (2018–2019)	44.65	3.94	17.25	1.52	−9.12_(383)_	<0.001
	TL2	Region	North	35.13	0.94	16.29	0.43	−20.59_(1172)_	<0.001
			Centre	27.69	3.75	15.51	2.09	−3.28_(1172)_	<0.01
			Alentejo	32.10	1.62	17.85	0.90	−9.91_(1172)_	<0.001
			Lisbon	32.55	2.99	17.85	0.90	−3.67_(1172)_	<0.001
			Setúbal	32.54	4.26	18.26	2.39	−3.38_(1172)_	<0.01
			Algarve	29.84	2.19	19.32	1.42	−4.72_(1172)_	<0.001
		WhySchool period	I (2015–2016)	24.07	0.90	15.28	0.57	−8.12_(1172)_	<0.001
			II (2018–2019)	41.37	2.54	21.20	1.30	−9.69_(1172)_	<0.001
	SHPs	WhySchool period	I (2015–2016)	18.27	1.35	11.28	0.83	−6.52_(394)_	<0.001
			II (2018–2019)	40.70	3.93	13.09	1.26	−8.93_(394)_	<0.001
Openness (%)	TL1	WhySchool period	I (2015–2016)	79.28	2.30	78.88	2.30	−0.19_(382)_	0.85
			II (2018–2019)	65.83	2.83	76.47	3.00	5.68_(382)_	<0.001
	TL2	Gender	Women	70.96	2.15	76.53	2.12	2.68_(1170)_	<0.01
			Men	60.83	2.79	73.36	2.89	4.56_(1170)_	<0.001
		Region	North	58.78	1.32	74.54	1.40	11.96_(1170)_	<0.001
			Centre	52.09	6.01	68.94	6.35	2.72_(1170)_	<0.01
			Alentejo	85.96	2.72	83.92	2.62	0.82_(1170)_	0.41
			Lisbon	76.59	4.58	86.87	4.68	2.39_(1170)_	<0.05
			Setúbal	54.16	5.88	64.65	6.03	1.77_(1170)_	<0.05
			Algarve	70.62	3.63	71.90	3.58	0.37_(1170)_	0.71
		WhySchool period	I (2015–2016)	56.48	1.85	71.54	1.96	8.07_(1170)_	<0.001
			II (2018–2019)	75.83	3.21	78.41	3.09	0.85_(1170)_	0.40
	SHPs	WhySchool period	I (2015–2016)	64.89	2.11	76.18	2.20	5.48_(395)_	<0.001
			II (2018–2019)	85.28	3.67	90.50	3.76	2.36_(395)_	<0.05
Confidence (2–20)	TL1	Age	≤ 39	6.95	0.79	14.54	0.99	9.10_(379)_	<0.001
			40–9	9.63	0.54	14.68	0.64	12.01_(379)_	<0.001
			≥ 50	10.80	0.54	14.73	0.61	9.46_(379)_	<0.001
		WhySchool period	I (2015–2016)	9.29	0.51	15.58	0.59	13.02_(379)_	<0.001
			II (2018–2019)	8.81	0.63	13.75	0.76	11.73_(379)_	<0.001
	SHPs	Age	≤ 39	9.45	0.58	14.47	0.68	9.27_(385)_	<0.001
			40–9	11.68	0.74	14.22	0.79	4.05_(385)_	<0.001
			≥50	11.11	0.79	14.14	0.87	4.19_(385)_	<0.001
		Gender	Women	13.38	0.58	15.13	0.61	3.74_(385)_	<0.001
			Men	8.37	0.74	13.45	0.88	7.02_(385)_	<0.001
		Region	North + Centre	10.52	0.47	13.02	0.50	5.91_(385)_	<0.001
			Alentejo	10.72	0.86	13.27	0.93	3.30_(385)_	<0.01
			Lisbon + Setúbal^a^	8.32	0.88	13.81	1.08	5.98_(385)_	<0.001
			Algarve	13.68	1.26	17.19	1.38	3.18_(385)_	<0.01
		WhySchool period	I (2015–2016)	10.72	0.57	13.15	0.60	4.94_(385)_	<0.001
			II (2018–2019)	10.73	0.76	15.45	0.89	6.73_(385)_	<0.001

MHK, mental health knowledge; pers DSS, personal Depression Stigma Subscale; GLMMs, generalised linear mixed models; Openness, openness to seeking help; Confidence, confidence in identifying or referring students; TL1, teachers Level 1; TL2, teachers Level 2; SHPs, school health professionals.

a. For Level 1 teachers and SHP groups, we merged the Centre region with the North and Setúbal with Lisbon to ensure statistical robustness, given the small number of participants in those areas.

SHPs achieved moderate but significant improvements, with WhySchool II participants showing the greatest gains. All age groups improved significantly, though participants aged 40 to 49 showed smaller increases. All regions demonstrated significant improvements. The youngest participants (39 or younger) demonstrated the most substantial improvements, while regional differences were also evident across implementation areas (Table 4).

The models explained 72.5% of the variance in MHK for Level 1 teachers, 62.6% for Level 2 teachers and 51.7% for SHPs.

### Personal depression stigma

Personal depression stigma decreased across all groups, with teachers at both levels demonstrating the greatest reductions and SHPs also achieving meaningful improvements (Table 3). WhySchool II participants showed the most substantial stigma reductions across all professional groups.

WhySchool I participants also achieved significant stigma reductions, demonstrating the intervention’s effectiveness across both implementation periods. Regional variations emerged among Level 2 teachers, with all regions showing significant reductions. The North region demonstrated the strongest improvements, while other regions including the Centre, Alentejo, Lisbon, Setúbal and the Algarve also achieved meaningful reductions, though with varying magnitudes (Table 4).

Among SHPs, both implementation periods showed significant improvements (Table 4).

All models explained a moderate variance in personal depression stigma (TL1 = 51.8%, TL2 = 53.9%, SHP = 59.8%).

### Openness to seeking help

Changes in openness to seeking help varied significantly across professional groups and implementation periods (Table 3). The overall pattern revealed differential responses that were influenced by participants’ baseline levels and the implementation context (Table 3).

Level 1 teachers showed no significant improvement overall in openness to seeking help. However, this masked differences between implementation periods (Table 3). WhySchool I participants maintained their already high baseline scores with no significant change (Table 4). In contrast, WhySchool II participants, who began with lower baseline openness, demonstrated statistically significant improvements (Table 4).

Level 2 teachers achieved significant overall improvements, though with demographic variations (Table 4). Both men and women showed meaningful gains, with men demonstrating greater improvements after starting from lower baseline levels. Regional differences were evident, with most areas showing significant improvements, except the Algarve and Alentejo regions, which had high scores on the baseline, but showed non-significant changes.

SHPs demonstrated significant improvements in openness in both implementation periods (Table 4). Both WhySchool I and WhySchool II participants achieved meaningful gains, indicating consistent positive changes across different contexts for this professional group.

All models explained a moderate variance in the outcome (TL1 = 43.4%, TL2 = 63.0%, SHP = 56.5%).

### Confidence in identifying or referring students potentially in need of help

Confidence increased across all groups, with teachers showing larger improvements than SHPs (Table 3). MHK partially accounted for the training effect for teachers.

Level 1 teachers demonstrated the largest overall gains across all demographic subgroups (Table 4). Age emerged as a significant factor, with older participants showing greater baseline confidence, though all age groups achieved significant improvements (Table 3). The implementation period also influenced outcomes, with WhySchool I participants showing slightly stronger improvements compared with WhySchool II participants.

Level 2 teachers showed widespread improvements (Table 3). MHK remained a significant predictor of confidence gains in this group.

SHPs showed more modest but consistent improvements across diverse participant characteristics (Table 4). Age, gender, regional location, prior training experience and the implementation period all emerged as significant factors influencing confidence outcomes (Table 3). While specific subgroups demonstrated stronger gains, including WhySchool II participants, men, younger participants and those in specific regions, all demographic categories achieved significant improvements.

All models explained a considerable variance in confidence (TL1 = 72.2%, TL2 = 61.8%, SHP = 69.6%).

### Training programme satisfaction

The participants’ evaluation of the training programme was highly positive, with all dimensions scoring above 4 (‘adequate’) in the 1-to-6 scale. Teachers reported a mean satisfaction score of 5.2 (s.d. = 0.66), with no significant difference between training levels (*t* = 0.95_(786),_ *p* = 0.35). SHPs rated the training slightly lower, with a mean of 4.83 (*F* = 16.97_(2)_, *p* < 0.001). Most participants considered the content relevance (teachers = 80.55%; SHPs = 62.3%), trainer quality (teachers = 91.75%; SHPs = 82.6%) and logistics (teachers = 78.3%; SHPs = 63.0%) as ‘very adequate’ or ‘completely adequate’.

Regarding overall satisfaction, most participants found it ‘very helpful’ or ‘extremely helpful’. There were no differences between teachers’ training level (χ^2^ = 8.15_(5)_, *p* = 0.09), but a significantly higher proportion of teachers expressed high satisfaction compared with SHPs (teachers = 82.6%, SHPs = 67.7%; χ^2^ = 26.98_(6)_, *p* = <0.001). No participants rated it as ‘not helpful at all,’ and only a minority considered it ‘somewhat helpful’ (teachers = 2.1%; SHPs = 6.5%).

## Discussion

This study evaluated a school-based MHL programme implemented among teachers and SHPs across mainland Portugal. Our findings demonstrate that this comprehensive intervention was associated with significant improvements in all four core components of MHL across both professional groups, including enhanced MHK, reduced personal stigma towards depression, increased openness to seeking help and greater confidence in identifying and referring students potentially in need of help. Notably, the extent of these changes varied across professional groups and demographic characteristics, with those having lower baseline scores generally benefiting most from the training.

### A promising intervention for a recognised need

Our findings underscore the critical need for MHL training within educational settings. The baseline assessment revealed moderate levels of MHK and confidence among both professional groups, despite relatively low stigma and favourable attitudes towards help-seeking. This pattern suggests that while educators may possess positive attitudes towards mental health, they lack the practical knowledge and confidence necessary to effectively support students, aligning with literature showing that knowledge deficits represent a key barrier to teachers’ mental health support provision.^6,23,29^

This need for enhanced competence was evident across the sample, although it manifested differently between the two professional groups. As expected, SHPs held significantly higher baseline scores in MHK and confidence, given their academic training and professional roles. Nevertheless, these were still only moderate, highlighting the importance of continuous training even for more specialised staff. For teachers, however, the challenge appeared more foundational, since the majority had never attended mental health training. This lack of training among educators highlights a potential gap in comprehensive professional development, leaving many educators feeling unprepared to address students’ mental health needs.^18–20^

### The WhySchool outcomes and professional group comparisons

The WhySchool training programme was associated with significant improvements in MHL across all measured domains in both teachers and SHPs. However, the magnitude of these gains varied across different professional groups and outcomes. Teachers demonstrated the largest effect size in MHK, a finding consistent with prior research, which reports that knowledge gains are often the most pronounced outcome of such interventions.^6,29^ Both professional groups showed substantial reductions in personal depression stigma. This may be attributable to their moderate baseline MHK, allowing for a greater focus on attitudinal change during the training. Previous studies show that while knowledge gains are often the most pronounced, shifts in stigmatising attitudes are also a key observed outcome of MHL programmes for professionals working with young people.^38^ In contrast, openness to seeking help showed a more modest improvement, yielding the smallest effect size across both professional groups. This suggests that help-seeking intentions may be more resistant to short-term change and therefore require longer follow-up to be properly assessed.^39^

Our findings suggest that the programme was particularly beneficial for participants starting with lower baseline competencies, effectively targeting those most in need of support. The differential in benefit was also reflected in the programme’s evaluation. Both professional groups reported high satisfaction, with teachers showing particularly positive evaluations. This likely reflects the higher perceived value and utility of the content for educators, for whom the material was more novel and directly addressed their most significant skill gaps.

### The ‘train-the-trainer’ model: a successful strategy for teachers

A particularly encouraging finding was the success of the cascade ‘train-the-trainer’ model. While the Level 1 teachers (TL1), a group of school leaders who received more intensive training, demonstrated substantial gains in MHK and confidence, what is most significant is the remarkable effectiveness observed for the Level 2 teachers (TL2). Despite receiving peer-led training and over a shorter period, the TL2s showed moderate to large significant improvements across all outcomes. These results suggest that despite the potential for a ‘message dilution’ effect often observed in such models,^40,41^ this approach is effective when properly implemented.

This success likely reflected several key implementation strategies. The WhySchool trainers, who were experienced MHPs with solid pedagogical and communication skills, provided high-quality preparation, supervision and ongoing support to TL1 facilitators. Structured materials, clear guidance and flexibility for contextual adaptation helped maintain training fidelity while ensuring local relevance. The high levels of programme satisfaction reported by the teachers, which notably did not differ between the two training levels, reinforce that participants perceived the intervention as valuable and well-executed, factors known to enhance engagement, mitigate the typical risks of the cascade model and ensure high-fidelity delivery.^42^

### The influence of demographics, context and the implementation period

The programme’s gains varied across different demographic and contextual factors. Our analysis revealed that training outcomes were influenced by participant characteristics, implementation context and the specific period during which the intervention was delivered. However, the replication of similar patterns of improvement across both implementation phases (WhySchool I and II) supports the robustness and reliability of the programme’s outcomes across different settings. Together, these findings underscore the importance of considering individual and contextual factors when implementing school-based MHL programmes, in line with previous research.^5,43^

### Strengths, limitations and future research

Some limitations should be considered. The convenience sampling method and the absence of a control group restrict the study’s ability to draw causal conclusions. Additionally, the post-test was only conducted one to two weeks after the training, providing no data on the long-term maintenance of observed improvements. Furthermore, while based on evidence-based frameworks, the MHK questionnaire’s validation used a single-sample approach, and WS I help-seeking openness relied on non-validated items before transitioning to a validated scale in WS II. Future research should implement random sampling methods with appropriate control groups and longitudinal follow-ups using consistent, validated instruments to ensure the comparability of findings and the real-world application of MHL programmes.

Despite these limitations, the study has several strengths that enhance the credibility and relevance of our findings. First, it is grounded in a sizable and diverse sample of 788 teachers and 201 SHPs (mainly psychologists) from multiple regions across mainland Portugal. The sample is also demographically representative: participants were predominantly female, reflecting the global trend in teaching^44^ and school psychology,^45^ and the average ages of both teachers (nearly 50) and SHPs (around 40) are typical for the Portuguese educational system.^45,46^ Second, the study provides a nuanced understanding of the intervention by comparing the two professional groups across two distinct implementation phases, offering valuable insights into their differential training needs and outcomes. Third, the use of a cascade ‘train-the-trainer’ model demonstrated a method for efficient and sustainable knowledge dissemination, empowering schools and fostering teacher-community support. Fourth, the programme targeted and measured not only knowledge and attitudes, but also professionals’ confidence to act.

In conclusion, the WhySchool programme resulted in observable improvements in the MHL of teachers and SHPs, including increased knowledge, reduced stigma, improved help-seeking attitudes and strengthened confidence to support students. Our findings yield two key strategic insights: the value of targeting professionals most in need of training, and the importance of tailoring interventions to individual and contextual needs. Moreover, the success of the cascade ‘train-the-trainer’ model confirms a viable pathway for sustainable, large-scale dissemination. These results underscore the importance of investing in school staff’s MHL as a foundational, evidence-based strategy for creating more supportive and mentally healthy educational communities.

## Supporting information

10.1192/bjo.2026.12009.sm001 Moreira et al. supplementary material Moreira et al. supplementary material

## Data Availability

The data are available upon reasonable request.

## Supplementary material

The supplementary material is available online at https://doi.org/10.1192/bjo.2026.12009

## Table 1 Long description

The table presents the characteristics of 989 participants categorized into three groups: 195 teachers at Level 1 (TL1), 593 teachers at Level 2 (TL2), and 201 school health professionals (SHPs), including 125 psychologists. The table has 12 rows and 5 columns. Column headers are Teachers Level 1 (TL1), Teachers Level 2 (TL2), School health professionals (SHPs), and Total. Row labels include Age, Age groups, Gender, School level, Teaching area, Professional category, Prior mental health training, and Implementation phase. Row 1: Age, M (s.d.), Teachers Level 1 (TL1), 48.15 (7.49); Teachers Level 2 (TL2), 48.14 (7.72); School health professionals (SHPs), 41.02 (9.06); Total, 46.69 (8.22). Row 2: Age groups, n (%)a, Teachers Level 1 (TL1), ≤39 years, 24 (12.3); 40–9 years, 86 (44.1); ≥50 years, 85 (43.6). Teachers Level 2 (TL2), ≤39 years, 103 (17.4); 40–9 years, 244 (41.1); ≥50 years, 265 (44.7). School health professionals (SHPs), ≤39 years, 109 (54.2); 40–9 years, 57 (28.4); ≥50 years, 35 (17.4). Total, ≤39 years, 236 (23.9); 40–9 years, 387 (39.1); ≥50 years, 385 (38.9). Row 3: Gender, n (%), Women, Teachers Level 1 (TL1), 155 (79.5); Teachers Level 2 (TL2), 473 (79.8); School health professionals (SHPs), 170 (84.6); Total, 798 (80.7). Men, Teachers Level 1 (TL1), 40 (20.5); Teachers Level 2 (TL2), 120 (20.2); School health professionals (SHPs), 31 (15.4); Total, 191 (19.3). Row 4: School level, n (%), Middle School (7th–9th), Teachers Level 1 (TL1), 117 (60.0); Teachers Level 2 (TL2), 368 (62.0); Total, 484 (61.3)b. High School (10th–12th), Teachers Level 1 (TL1), 78 (40.0); Teachers Level 2 (TL2), 225 (38.0); Total, 303 (38.5)b. Row 5: Teaching area, n (%), Sciences, Teachers Level 1 (TL1), 98 (50.3); Teachers Level 2 (TL2), 247 (41.7); Total, 345 (43.8)b. Humanities/Social Sciences, Teachers Level 1 (TL1), 96 (49.2); Teachers Level 2 (TL2), 343 (57.8); Total, 439 (55.7)b. NA, Teachers Level 1 (TL1), 1 (0.5); Teachers Level 2 (TL2), 3 (0.5); Total, 4 (0.5)b. Row 6: Professional category, n (%), Psychologists, School health professionals (SHPs), 125 (62.2); Total, 125 (12.6). Other SHPs, School health professionals (SHPs), 76 (37.8); Total, 76 (7.7). Row 7: Prior mental health training, n (%), No, Teachers Level 1 (TL1), 138 (70.8); Teachers Level 2 (TL2), 476 (80.3); School health professionals (SHPs), 35 (17.4); Total, 649 (65.6). Yes, Teachers Level 1 (TL1), 57 (29.2); Teachers Level 2 (TL2), 117 (19.7); School health professionals (SHPs), 166 (82.6); Total, 340 (34.4). Row 8: Implementation phase, n (%), WhySchool I (2015–2016), Teachers Level 1 (TL1), 87 (44.6); Teachers Level 2 (TL2), 411 (69.3); School health professionals (SHPs), 104 (51.7); Total, 602 (60.9). WhySchool II (2018–2020), Teachers Level 1 (TL1), 108 (55.4); Teachers Level 2 (TL2), 182 (30.7); School health professionals (SHPs), 97 (48.3); Total, 387 (39.1).

Navigate back to Table 1.

## Table 2 Long description

The table presents a comparison of baseline and post-training outcomes across different professions and training levels. It includes data for MHK, pers DSS, openness, and confidence. The table has 12 rows and 8 columns. The columns are labeled as Baseline, Post-training, and Cohen's d, with sub-columns for M (s.d.), t(df), p, Point estimate, and 95% CI. The rows are labeled with different outcomes and subcategories such as MHK (%), TL1, TL2, SHPs, pers DSS (%), Openness (%), and Confidence (2-20). Each row provides specific values for the baseline and post-training periods, along with statistical measures like t(df), p, Point estimate, and 95% CI. Notable trends include significant improvements in MHK and reductions in pers DSS across all groups, with varying effect sizes indicated by Cohen's d.

Navigate back to Table 2.

## Table 3 Long description

The table presents the results of generalised linear mixed models for various outcome scores by profession and training level. It includes columns for B, S E, t, p, and 95 percent CI. The table is divided into sections for different outcome scores: MHK, pers DSS, Openness, and Confidence. Each section is further divided by training level (TL 1, TL 2, SHPs) and includes rows for intercept, time, gender, and various regions and post-training interactions. The table lists specific values for each factor, such as intercept, time, gender, and regions like Algarve, Lisbon, Alentejo, and Centro. Notable trends include significant p-values and varying B values across different factors and interactions.

Navigate back to Table 3.

## Table 4 Long description

The table presents data on estimated means, standard errors, and paired analysis for various outcomes at baseline and post-training. It includes columns for professional group, factor, level, baseline estimated mean, baseline standard error, post-training estimated mean, post-training standard error, t-value, and p-value. The outcomes measured are MHK (%), pers DSS (%), openness (%), and confidence (2-20). The table is divided into sections for TL1, TL2, and SHPs, with various factors such as gender, region, prior training, age, and WhySchool period being analyzed. Each row provides specific data points for these factors, showing the changes from baseline to post-training. Notable trends include significant improvements in various regions and groups, with detailed statistical analysis provided for each outcome.

Navigate back to Table 4.
