# Supplementary material for: WhySchool project: effects of a school-based mental health literacy programme on teachers and school health professionals
Source: BJPsych Open. 2026 Jul 6;12(4):e175. doi: 10.1192/bjo.2026.12009 (PMC13359052; doi:10.1192/bjo.2026.12009)
Supplement: Moreira et al. supplementary material [file S2056472426120092sup001.docx]

**WhySchool Project: Effects of a School-Based Mental Health Literacy Programme on Teachers and School Health Professionals**

**Supplementary File**

1. **WhySchool training programme**

Table S1- Structure and Content of the Intervention Curriculum

| **Theme** | **Module** |
| --- | --- |
| Adolescence and Mental Life | 1. Adolescence and typical development |
|  | 1. Brain’s complex functions |
|  | 1. The “normal” limits |
| The Burden of Mental Disorders | 1. Mental illness in young people |
|  | 1. Mental health stigma |
| Behavioural Problems and Mental Disorders | 1. Behaviour problems and mental disorders |
|  | 1. Suicidal and self-harm behaviours |
| Interventions | 1. Treatment and Interventions |
|  | 1. Communication and Confidentiality |
| Mental Health Promotion | 1. Healthy Lifestyles |
|  | 1. Preparing a lesson |
| Other Topics | 1. Bullying – harassment and violence among young people in school |

1. **Mental Health Knowledge (MHK) questionnaire**

Table S2 - Psychometric properties of the final 31-item Mental Health Knowledge questionnaire (one-factor model) for Teachers and School Health Professionals (n= 989 participants from WhySchool I and II)

| **Analysis** | **Parameter** | **Value** |
| --- | --- | --- |
| **Exploratory Factor Analysis** | One factor explained variance | 20.0% |
|  | Factor loading | 0.24 – 0.89^a^ |
| **Internal Consistency** | Cronbach’s Alpha | 0.89 |
|  | McDonald's Omega | 0.92 |
| **Confirmatory Factor Analysis** | Factor loading | 0.18 - 0.83^b^ |
|  | CFI | 0.88 |
|  | TLI | 0.87 |
|  | RMSEA | 0.06 |
|  | SRMR | 0.12 |
| CFI = Comparative Fit Index; TLI = Tucker-Lewis Index; RMSEA = Root Mean Square Error of Approximation; SRMR = Standardized Root Mean Square Residual. ^a^ For further details, see Table S3; ^b^ For further details, see Table S4. *Note: Low factor loadings and explained variance are consistent with the formative nature of knowledge-based assessments, where items cover heterogeneous facts rather than a single latent trait. Content validity was prioritized to ensure full curricular coverage. Despite some item heterogeneity, the model demonstrated adequate CFA fit indices, and scale reliability is robustly supported by both Cronbach’s Alpha and McDonald’s Omega.* | | |

Table S3 - Exploratory Factor Analysis of the Mental Health Knowledge questionnaire: item description and factor loadings.

| **Item** | **Factor loading** |
| --- | --- |
| 14. Na depressão podem ocorrer pensamentos suicidas. | 0.85 |
| 11. Problemas socio culturais e doença mental podem ambos contribuir para as dificuldades da aprendizagem. | 0.81 |
| 30. É melhor falar com um pai por telefone sobre o comportamento dos seus filhos do que presencialmente. | 0.75 |
| 26. *Bullying* persistente pode levar a muitas emoções negativas e até mesmo à recusa da escola em alguns adolescentes. | 0.74 |
| 10. As pessoas que sofrem de uma doença mental nunca recuperam. | 0.57 |
| 13. Pode ser necessário adaptar o currículo para alunos que têm uma doença mental. | 0.57 |
| 22. O suicídio nos adolescentes resulta geralmente do stress de ser adolescente. | 0.56 |
| 16. A ansiedade é uma resposta normal perante o stress de exames escritos. | 0.55 |
| 3. O ambiente tem pouco ou nenhum impacto no desenvolvimento do cérebro. | 0.50 |
| 6. Uma doença mental é causada por uma disfunção cerebral levando a problemas de adaptação. | 0.48 |
| 19. TCC – A terapia cognitivo-comportamental geralmente não é eficaz no tratamento de jovens com perturbações da ansiedade. | 0.47 |
| 23. Uma tentativa de suicídio anterior é o fator de risco mais elevado para o suicídio num adolescente. | 0.46 |
| 2. Mudanças no desenvolvimento do cérebro explicam muitas das características da adolescência. | 0.45 |
| 8. As doenças mentais contribuem para o maior peso de doença nos adolescentes em todo o mundo. | 0.45 |
| 7. A maioria das doenças mentais podem ser diagnosticadas antes dos 25 anos. | 0.44 |
| 32. As pessoas com doença mental são mais frequentemente vítimas do que perpetradoras de violência. | 0.42 |
| 28. Todos os tratamentos eficazes da doença mental mudam a forma como o cérebro funciona. | 0.41 |
| 31. Saúde mental é saúde do cérebro. | 0.40 |
| 20. Compulsões são pensamentos obsessivos sobre germes. | 0.40 |
| 4. Percepção, cognição, e afectividade são todas funções do cérebro. | 0.40 |
| 33. Os professores devem ignorar histórias estigmatizantes sobre doença mental que aparecem nos *Media*. | 0.36 |
| 9. Estigma e discriminação são a mesma coisa. | 0.35 |
| 18. Os ataques de pânico são sempre resultado de uma perturbação de pânico. | 0.34 |
| 24. Um professor deve sempre tentar determinar se um aluno que parece deprimido é suicida. | 0.32 |
| 34. Se um aluno na escola morrer por suicídio a escola deve organizar uma assembleia com todos os alunos para discutir o que aconteceu. | 0.29 |
| 25. Muitos adolescentes que são vítimas de *bullying* podem ter uma doença mental pré-existente. | 0.29 |
| 12. A ilusão é uma alucinação que ocorre durante uma psicose. | 0.27 |
| 1. O cérebro humano está praticamente desenvolvido por volta dos 10 anos. | 0.26 |
| 27. Tratamentos alternativos são geralmente muito eficazes para as pessoas que têm uma doença mental. | 0.25 |
| 17. A melhor forma de um professor ajudar um aluno que tem uma perturbação de ansiedade é protegê-lo do stress. | 0.24 |
| 15. A hospitalização é frequentemente indicada no tratamento da mania. | 0.24 |

Table S4- Confirmatory Factor Analysis of Mental Health Knowledge questionnaire: item description and standardised factor loadings.

| **Item** | **Factor loading** |
| --- | --- |
| 14. Na depressão podem ocorrer pensamentos suicidas. | 0.98 |
| 26. *Bullying* persistente pode levar a muitas emoções negativas e até mesmo à recusa da escola em alguns adolescentes. | 0.91 |
| 11. Problemas socio culturais e doença mental podem ambos contribuir para as dificuldades da aprendizagem. | 0.83 |
| 30. É melhor falar com um pai por telefone sobre o comportamento dos seus filhos do que presencialmente. | 0.79 |
| 10. As pessoas que sofrem de uma doença mental nunca recuperam. | 0.64 |
| 13. Pode ser necessário adaptar o currículo para alunos que têm uma doença mental. | 0.63 |
| 19. TCC – A terapia cognitivo-comportamental geralmente não é eficaz no tratamento de jovens com perturbações da ansiedade. | 0.61 |
| 22. O suicídio nos adolescentes resulta geralmente do stress de ser adolescente. | 0.60 |
| 16. A ansiedade é uma resposta normal perante o stress de exames escritos. | 0.58 |
| 28. Todos os tratamentos eficazes da doença mental mudam a forma como o cérebro funciona. | 0.54 |
| 18. Os ataques de pânico são sempre resultado de uma perturbação de pânico. | 0.53 |
| 20. Compulsões são pensamentos obsessivos sobre germes. | 0.53 |
| 3. O ambiente tem pouco ou nenhum impacto no desenvolvimento do cérebro. | 0.52 |
| 7. A maioria das doenças mentais podem ser diagnosticadas antes dos 25 anos. | 0.50 |
| 8. As doenças mentais contribuem para o maior peso de doença nos adolescentes em todo o mundo. | 0.49 |
| 23. Uma tentativa de suicídio anterior é o fator de risco mais elevado para o suicídio num adolescente. | 0.47 |
| 2. Mudanças no desenvolvimento do cérebro explicam muitas das características da adolescência. | 0.46 |
| 12. A ilusão é uma alucinação que ocorre durante uma psicose. | 0.46 |
| 25. Muitos adolescentes que são vítimas de *bullying* podem ter uma doença mental pré-existente. | 0.43 |
| 6. Uma doença mental é causada por uma disfunção cerebral levando a problemas de adaptação. | 0.42 |
| 4. Percepção, cognição, e afectividade são todas funções do cérebro. | 0.41 |
| 9. Estigma e discriminação são a mesma coisa. | 0.41 |
| 32. As pessoas com doença mental são mais frequentemente vítimas do que perpetradoras de violência. | 0.40 |
| 17. A melhor forma de um professor ajudar um aluno que tem uma perturbação de ansiedade é protegê-lo do stress. | 0.38 |
| 15. A hospitalização é frequentemente indicada no tratamento da mania. | 0.38 |
| 33. Os professores devem ignorar histórias estigmatizantes sobre doença mental que aparecem nos *Media*. | 0.37 |
| 34. Se um aluno na escola morrer por suicídio a escola deve organizar uma assembleia com todos os alunos para discutir o que aconteceu. | 0.35 |
| 31. Saúde mental é saúde do cérebro. | 0.36 |
| 1. O cérebro humano está praticamente desenvolvido por volta dos 10 anos. | 0.36 |
| 24. Um professor deve sempre tentar determinar se um aluno que parece deprimido é suicida. | 0.33 |
| 27. Tratamentos alternativos são geralmente muito eficazes para as pessoas que têm uma doença mental. | 0.18 |

1. ***Missing data***

Table S5 - Number of participants and proportion of dropouts in each evaluation moment

|  |  | **Teachers Level 1 (TL1)**  **n=195** | | **Teachers Level 2 (TL2)**  **n=593** | | **School Health Professionals (SHPs)**  **n=201** | |
| --- | --- | --- | --- | --- | --- | --- | --- |
|  | **Variable** | **N** | **Missing (n, %)** | **N** | **Missing (%)** | **N** | **Missing (%)** |
| **t0** | Age | 195 | 0 (0.0) | 585 | 8 (1.3) | 201 | 0 (0.0) |
|  | Sex | 195 | 0 (0.0) | 593 | 0 (0.0) | 199 | 2 (1.0) |
|  | School Region | 195 | 0 (0.0) | 593 | 0 (0.0) | 201 | 0 (0.0) |
|  | Teaching area | 192 | 3 (1.5) | 537 | 56 (9.4) | - | - |
|  | School level | 163 | 32 )16.4) | 558 | 35 (5.9) | - | - |
|  | Professional category | - | - | - | - | 201 | 0 (0.0) |
|  | Previous MH training | 192 | 3 (1.5) | 593 | 0 (0.0) | 200 | 1 (0.5) |
|  | MHK | 192 | 3 (1.5) | 593 | 0 (0.0) | 200 | 1 (0.5) |
|  | pers DSS | 192 | 3 (1.5) | 592 | 1 (0.2) | 200 | 1 (0.5) |
|  | Openness | 192 | 3 (1.5) | 593 | 0 (0.0) | 200 | 1 (0.5) |
|  | Confidence | 192 | 3 (1.5) | 593 | 0 (0.0) | 200 | 1 (0.5) |
| **t1** | MHK | 186 | 9 (4.6) | 423 | 170 (28.7) | 140 | 61 (30.3) |
|  | pers DSS | 186 | 9 (4.6) | 423 | 170 (28.7) | 140 | 61 (30.3) |
|  | Openness | 186 | 9 (4.6) | 423 | 170 (28.7) | 140 | 61 (30.3) |
|  | Confidence | 186 | 9 (4.6) | 423 | 170 (28.7) | 140 | 61 (30.3) |
|  | Training satisfaction | 128 | 67 (34.4) | 324 | 269 (45.4) | 87 | 114 (56.7) |
| N = absolute number; t0= baseline evaluation; t1= post-training evaluation; MH = mental health; MHK = Mental Health Knowledge; pers DSS = personal Depression Stigma Subscale; Openness= Openness to seeking help; Confidence= confidence in identifying or referring students; | | | | | | | |

***Statistical Analysis***

We applied multiple imputations using the mice package in R to address missing data while preventing temporal data leakage. For both WhySchool I and II, we systematically identified time-sensitive variables using the grepl() function, ensuring that post-evaluation data did not inform pre-evaluation imputations. We constructed a predictor matrix with the quickpred() function, incorporating demographic, professional, and training-related variables while excluding highly collinear predictors (r>0.80). We assigned imputation methods according to variable type: predictive mean matching for continuous variables, logistic regression for binary variables, and polytomous regression for categorical variables. We performed five imputations with 10 iterations per imputation, following best-practice recommendations for ensuring stable and unbiased imputations.

***Results***

In both WhySchool I and II, we evaluated the quality of imputations based on convergence diagnostics, relative efficiency, distributional integrity, and model fit. However, due to differences in the volume and patterns of missing data, slightly different diagnostic strategies were applied.

In WS I, where the proportion of missing data was low and exploratory analysis indicated no major deviations from randomness, we selected the third imputed dataset as the final. It demonstrated excellent convergence (Brooks-Gelman-Rubin diagnostic=1.03), achieved a high relative efficiency (97.5%), and maintained distributional integrity, with Kolmogorov-Smirnov tests confirming no significant differences between imputed and observed data (p>0.05). Lower AIC values for imputed models indicated better model fit than those based on incomplete data.

In WSl II, where the proportion and structure of missingness were more complex, we conducted an additional assessment of missing data using the md.pattern() function, and Little’s MCAR test indicated that the data were not missing completely at random, which supports the use of multiple imputations. We selected the fourth imputed dataset, which met the same convergence and efficiency criteria as WS I (Brooks-Gelman-Rubin diagnostic=1.03, relative efficiency=97%). The Kolmogorov-Smirnov tests again revealed no significant distributional differences (p>0.05), and the AIC reduction (ΔAIC=-452.01) signified an improvement in model fit and data completeness.

1. **Participants’ characteristics and outcome scores at baseline**

Table S6 - Participants’ characteristics (Section A) and baseline outcome scores according to personal characteristics (Section B) by profession, training level and WhySchool period.

|  | | **Section A – Participants’ characteristics** | | | | | | | | | | | | | | | | | |  |
| --- | --- | --- | --- | --- | --- | --- | --- | --- | --- | --- | --- | --- | --- | --- | --- | --- | --- | --- | --- | --- |
|  | | **Teachers Level 1 (TL1)** | | | | **Teachers Level 2 (TL2)** | | | | | | | | | | **School Health Professionals (SHPs)** | | | |  |
|  | | **Total** | **WS I** | **WS II** | **p** | **Total** | | | **WS I** | | | **WS II** | | | **p** | **Total** | **WS I** | **WS II** | **p** |  |
| **Sample, n (%)** | | 195 (19.7) | 87 (14.5) | 108 (27.9) | **<0.001** | 593 (60.0) | | | 411 (68.3) | | | 182 (47.0) | | | **<0.001** | 201 (20.3) | 104 (17.3) | 97 (25.1) | **<0.01** |  |
| **Age, M(SD)** | | 48.15 (0.49) | 48.77 (6.12) | 47.65 (7.38) | 0.13 | 48.14 (0.31) | | | 47.00 (7.05) | | | 50.71 (7.66) | | | **<0.001** | 41.02 (0.65) | 39.46 (9.36) | 42.69 (8.68) | **<0.01** |  |
| **Age, n(%**)^a^ | ≤ 39 | 24 (12.3) | 5 (5.7) | 19 (17.6) | **<0.05** | 103 (17.4) | 79 (19.2) | | | 24 (13.2) | | | 0.07 | | | 109 (54.2) | 64 (61.5) | 45 (46.4) | **<0.05** |  |
|  | 40-49 | 86 (44.1) | 43 (49.4) | 43 (39.8) | 0.18 | 244 (41.1) | 187 (45.5) | | | 57 (31.3) | | | **<0.01** | | | 57 (28.4) | 19 (18.3) | 38 (39.2) | **<0.01** |  |
|  | ≥ 50 | 85 (43.6) | 39 (44.8) | 46 (42.6) | 0.75 | 265 (44.7) | 156 (38.0) | | | 109 (59.9) | | | **<0.001** | | | 42 (20.9) | 23 (22.1) | 19 (19.6) | 0.66 |  |
| **Sex, n(%)** | Women | 155 (79.5) | 72 (82.8) | 83 (76.9) | 0.31 | 473 (79.8) | 341 (83.0) | | | 132 (72.5) | | | **<0.01** | | | 170 (84.6) | 96 (92.3) | 74 (76.3) | **<0.01** |  |
|  | Men | 40 (20.5) | 15 (17.2) | 25 (23.1) |  | 120 (20.2) | 70 (17.0) | | | 50 (27.5) | | |  |  |  | 31 (15.4) | 8 (7.7) | 23 (23.7) |  |  |
| **School Region, n(%)** | North | 140 (71.8) | 32 (36.8) | 108 (100) |  | 343 (57.8) | 161 (39.2) | | | 182 (100) | | |  | | | 140 (69.7) | 43 (41.3) | 97 (100) |  |  |
|  | Centre | 5 (2.6) | 5 (5.7) | - |  | 14 (2.4) | 14 (3.4) | | | - | | |  | | | 2 (1.0) | 2 (1.9) | - |  |  |
|  | Alentejo | 31 (15.9) | 31 (35.6) | - |  | 136 (22.9) | 136 (33.1) | | | - | | |  | | | 28 (13.9) | 28 (26.9) | - |  |  |
|  | Lisbon | 8 (4.1) | 8 (9.2) | - |  | 32 (5.4) | 32 (7.8) | | | - | | |  | | | 17 (8.5) | 17 (16.3) | - |  |  |
|  | Setúbal | 1 (0.5) | 1 (1.1) | - |  | 15 (2.5) | | 15 (3.6) | | | - | | |  | | 1 (0.5) | 1 (1.0) | - |  |  |
|  | Algarve | 10 (5.1) | 10 (11.5) | - |  | 53 (8.9) | | 53 (12.9) | | | - | | |  | | 13 (6.5) | 13 (12.5) | - |  |  |
| **Teaching area, n(%)** | Sciences | 98 (50.3) | 41 (47.1) | 57 (52.8) | 0.46 | 247 (41.7) | | 158 (38.4) | | | 89 (48.9) | | | **<0.01** | | - | - | - |  |  |
|  | Human. | 96 (49.2) | 46 (52.9) | 50 (46.3) |  | 343 (57.8) | 253 (61.6) | | | 90 (49.5) | | |  | | | - | - | - |  |  |
|  | NA | 1 (0.5) | 0 | 1 (0.9) |  | 3 (0.5) | | 0 | | | 3 (1.6) | | |  | | - | - | - |  |  |
| **School level, n(%)** | 7^th^-9^th^ | 117 (60.0) | 51 (58.6) | 66 (61.1) | 0.72 | 368 (62.0) | | 246 (59.9) | | | 121 (66.5) | | | 0.09 | | - | - | - |  |  |
|  | 10^th^ -12^th^ | 78 (40.0) | 36 (41.4) | 42 (38.9) |  | 225 (38.0) | | 165 (40.1) | | | 61 (33.5) | | |  | | - | - | - |  |  |
| **Prior MH training** | No | 138 (70.8) | 59 (67.8) | 79 (73.1) | 0.42 | 476 (80.3) | | 343 (83.5) | | | 133 (73.1) | | | **<0.01** | | 35 (17.4) | 22 (21.2) | 13 (13.4) | 0.15 |  |
|  | Yes | 57 (29.2) | 28 (32.2) | 29 (26.9) |  | 117 (19.7) | | 68 (16.5) | | | 49 (26.8) | | |  |  | 166 (82.6) | 82 (78.8) | 84 (86.6) |  |  |
|  | | **Section B – Baseline outcome scores** | | | | | | | | | | | | | | | | | |  |
|  | | **Teachers Level 1 (TL1)** | | | | **Teachers Level 2 (TL2)** | | | | | | | | | | **School Health Professionals (SHPs)** | | | |  |
|  | | **Total** | **WS I** | **WS II** | **p** | **Total** | | **WS I** | | | **WS II** | | | **p** | | **Total** | **WS I** | **WS II** | **p** |  |
| **MHK M(SD)** | | 54.13 (16.13) | 55.36 (14.61) | 53.14 (17.26) | 0.34 | 52.80 (14.73) | | 53.43 (13.47) | | | 51.38 (17.21) | | | 0.12 | | 62.96 (21.16) | 63.90 (13.82) | 61.96 (26.95) | 0.52 |  |
| Age | ≤ 39 | 54.30 (22.60) | 55.48 (15.20) | 53.99 (24.52) | 0.90 | 52.46 (17.05) | 55.12 (15.47) | | | 41.13 (19.23) | | | **<0.01** | | | 61.04 (22.61) | 64.00 (13.66) | 56.45 (31.57) | 0.10 |  |
|  | 40-49 | 53.19 (16.73) | 55.74 (13.71) | 50.64 (19.12) | 0.16 | 51.64 (14.88) | 52.63 (13.08) | | | 48.39 (19.44) | | | 0.06 | | | 67.01 (21.65) | 67.74 (14.70) | 66.64 (24.57) | 0.86 |  |
|  | ≥ 50 | 55.03 (13.26) | 54.92 (15.83) | 55.12 (10.79) | 0.95 | 53.98 (13.75) | 53.64 (13.00) | | | 54.46 (14.78) | | | 0.64 | | | 62.14 (15.86) | 60.47 (13.19) | 64.18 (18.77) | 0.47 |  |
|  | p | 0.76 | 0.97 | 0.46 |  | 0.20 | 0.41 | | | **<0.01** | | |  | | | 0.23 | 0.24 | 0.23 |  |  |
| Sex | Women | 56.34 (12.91) | 54.84 (14.57) | 57.64 (10.53) | 0.17 | 53.88 (13.55) | 53.94 (13.23) | | | 53.72 (14.41) | | | 0.87 | | | 63.27 (17.93) | 63.28 (14.03) | 63.25 (22.10) | 0.99 |  |
|  | Men | 45.56 (23.92) | 57.85 (15.06) | 38.19 (25.43) | **<0.05** | 48.55 (18.13) | 50.92 (14.44) | | | 45.23 (22.03) | | | 0.09 | | | 61.29 (32.20) | 71.37 (8.35) | 57.78 (39.03) | 0.13 |  |
|  | p | **<0.001** | 0.47 | **<0.001** |  | **<0.001** | 0.09 | | | **<0.01** | | |  | | | 0.63 | 0.11 | 0.40 |  |  |
| Prior MH training | No | 55.12 (14.21) | 54.67 (15.44) | 55.45 (13.30) | 0.75 | 51.97 (13.91) | 52.41 (12.99) | | | 50.81 (16.03) | | | 0.26 | | | 51.89 (23.00) | 54.69 (17.40) | 47.15 (30.50) | 0.36 |  |
|  | Yes | 51.73 (19.98) | 56.80 (12.82) | 46.83 (24.27) | 0.06 | 56.19 (17.36) | 58.54 (14.75) | | | 52.94 (20.15) | | | 0.08 | | | 65.30 (20.06) | 66.37 (11.62) | 64.25 (25.80) | 0.50 |  |
|  | p | 0.18 | 0.53 | **<0.05** |  | **<0.01** | **<0.001** | | | 0.46 | | |  | | | **<0.01** | **<0.001** | **<0.05** |  |  |
| **pers DSS M(SD)** | | 36.64 (14.78) | 26.88 (11.50) | 44.50 (12.24) | **<0.001** | 31.58 (15.00) | | | 25.56 (12.55) | | | 45.16 (10.55) | | | **<0.001** | 33.13 (17.71) | 22.17 (15.64) | 44.87 (11.05) | **<0.001** |  |
| Age | ≤ 39 | 41.44 (16.48) | 25.00 (9.42) | 45.76 (15.25) | **<0.01** | 28.87 (13.79) | 24.88 (11.74) | | | 45.83 (7.59) | | | **<0.001** | | | 31.10 (17.26) | 23.21 (16.79) | 43.33 (8.85) | **<0.001** |  |
|  | 40-49 | 35.79 (14.06) | 28.29 (12.81) | 43.28 (11.00) | **<0.001** | 29.45 (14.32) | 24.63 (11.72) | | | 45.27 (10.10) | | | **<0.001** | | | 37.33 (14.49) | 18.57 (11.64) | 46.71 (11.22) | **<0.001** |  |
|  | ≥ 50 | 36.14 (14.92) | 25.57 (10.22) | 45.11 (12.17) | **<0.001** | 34.39 (15.54) | 26.98 (13.75) | | | 45.01 (11.21) | | | **<0.001** | | | 32.34 (18.55) | 22.34 (15.43) | 44.44 (14.49) | **<0.001** |  |
|  | p | 0.24 | 0.53 | 0.70 |  | **<0.001** | 0.20 | | | 0.95 | | |  | | | 0.10 | 0.53 | 0.40 |  |  |
| Sex | Women | 36.83 (14.16) | 27.28 (9.93) | 45.11 (11.89) | **<0.01** | 30.62 (14.99) | 24.98 (12.54) | | | 45.18 (10.28) | | | **<0.001** | | | 33.06 (18.26) | 22.95 (15.72) | 46.17 (11.90) | **<0.001** |  |
|  | Men | 35.90 (17.16) | 25.00 (17.57) | 42.44 (13.41) | **<0.01** | 35.35 (14.48) | 28.37 (12.32) | | | 45.11 (11.33) | | | **<0.001** | | | 33.51 (14.65) | 12.85 (11.78) | 40.70 (6.25) | **<0.001** |  |
|  | p | 0.73 | 0.49 | 0.34 |  | **<0.01** | **<0.05** | | | 0.97 | | |  | | | 0.90 | 0.08 | **<0.05** |  |  |
| Prior MH training | No | 36.63 (15.24) | 26.41 (12.16) | 44.27 (12.65) | **<0.001** | 31.00 (15.05) | 25.40 (12.75) | | | 45.43 (10.14) | | | **<0.001** | | | 32.14 (14.89) | 25.38 (13.99) | 43.59 (7.72) | **<0.001** |  |
|  | Yes | 36.65 (13.73) | 27.88 (10.13) | 45.11 (11.28) | **<0.001** | 33.93 (14.62) | 26.35 (11.55) | | | 44.44 (11.66) | | | **<0.001** | | | 33.33 (18.29) | 21.31 (16.02) | 45.07 (11.50) | **<0.001** |  |
|  | p | 0.99 | 0.58 | 0.75 |  | 0.06 | 0.57 | | | 0.58 | | |  | | | 0.68 | 0.28 | 0.66 |  |  |
| **Openness M(SD)** | | 76.25 (18.98) | 83.77 (17.27) | 70.19 (18.17) | **<0.001** | 65.85 (26.38) | | | 63.72 (28.21) | | | 70.66 (20.98) | | | **<0.01** | 68.95 (22.86) | 61.78 (26.41) | 76.63 (15.00) | **<0.001** |  |
| Age | ≤ 39 | 68.23 (23.08) | 87.50 (12.50) | 63.16 (22.70) | **<0.05** | 66.10 (29.64) | 64.89 (30.79) | | | 71.25 (24.31) | | | 0.44 | | | 68.00 (24.15) | 63.91 (27.27) | 74.33 (16.71) | **<0.05** |  |
|  | 40-49 | 77.78 (17.30) | 85.17 (15.25) | 70.39 (16.15) | **<0.001** | 63.80 (26.65) | 64.10 (27.42) | | | 62.81 (24.17) | | | 0.75 | | | 75.98 (19.36) | 65.13 (26.87) | 81.40 (11.19) | **<0.01** |  |
|  | ≥ 50 | 76.96 (19.03) | 81.74 (19.84) | 72.90 (17.52) | **<0.05** | 67.65 (24.97) | 62.74 (28.13) | | | 74.68 (17.42) | | | **<0.001** | | | 61.71 (21.83) | 53.26 (22.68) | 71.93 (15.96) | **<0.01** |  |
|  | p | 0.08 | 0.60 | 0.14 |  | 0.26 | 0.84 | | | **<0.01** | | |  | | | **<0.01** | 0.21 | **<0.05** |  |  |
| Sex | Women | 77.87 (18.73) | 85.60 (16.61) | 71.16 (17.95) | **<0.01** | 67.96 (26.18) | 65.69 (28.20) | | | 73.84 (18.90) | | | **<0.01** | | | 68.20 (23.16) | 60.29 (25.90) | 78.47 (13.40) | **<0.001** |  |
|  | Men | 69.96 (18.86) | 75.00 (18.30) | 66.93 (18.90) | 0.19 | 57.51 (25.59) | 54.11 (26.39) | | | 62.27 (23.89) | | | 0.08 | | | 73.04 (20.99) | 79.69 (27.50) | 70.72 (18.39) | 0.41 |  |
|  | p | **<0.05** | **<0.05** | **0.31** |  | **<0.001** | **<0.01** | | | **<0.01** | | |  | | | 0.28 | 0.05 | **<0.05** |  |  |
| Prior MH training | No | 76.59 (18.65) | 85.59 (15.19) | 69.87 (18.24) | **<0.001** | 65.44 (26.49) | 63.41 (28.19) | | | 70.68 (20.71) | | | **<0.01** | | | 62.33 (22.42) | 53.41 (20.84) | 77.44 (16.45) | **<0.01** |  |
|  | Yes | 75.40 (19.89) | 79.93 (20.80) | 71.03 (18.28) | 0.09 | 67.50 (25.96) | 65.26 (28.48) | | | 70.61 (21.90) | | | 0.27 | | | 70.34 (22.77) | 64.02 (27.39) | 76.51 (14.87) | **<0.001** |  |
|  | p | 0.69 | 0.15 | 0.77 |  | 0.45 | 0.62 | | | 0.99 | | |  | | | 0.06 | 0.09 | 0.84 |  |  |
| **Confidence M(SD)** | | 9.33 (4.32) | 9.98 (4.12) | 8.81 (4.41) | 0.06 | 9.29 (3.86) | | | 9.26 (3.87) | | | 9.36 (3.86) | | | 0.76 | 13.02 (4.93) | 13.13 (4.30) | 12.91 (5.54) | 0.76 |  |
| Age | ≤ 39 | 6.42 (3.05) | 8.60 (1.67) | 5.84 (3.10) | 0.07 | 9.61 (4.12) | 10.07 (4.08) | | | 7.63 (3.78) | | | **<0.05** | | | 12.18 (5.37) | 13.15 (4.54) | 10.68 (6.22) | **<0.05** |  |
|  | 40-49 | 9.19 (4.09) | 9.60 (4.19) | 8.77 (4.00) | 0.35 | 9.15 (3.60) | 9.38 (3.57) | | | 8.39 (3.61) | | | 0.07 | | | 13.96 (4.86) | 13.68 (4.42) | 14.11 (5.11) | 0.76 |  |
|  | ≥ 50 | 10.31 (4.49) | 10.56 (4.25) | 10.09 (4.71) | 0.31 | 9.32 (4.02) | 8.76 (4.06) | | | 10.13 (3.83) | | | **<0.01** | | | 13.79 (3.39) | 12.61 (3.58) | 15.21 (2.57) | **<0.05** |  |
|  | p | **<0.001** | 0.43 | **<0.01** |  | 0.63 | 0.05 | | | **<0.01** | | |  | | | 0.05 | 0.72 | **<0.01** |  |  |
| Sex | Women | 9.63 (4.38) | 9.82 (4.40) | 9.47 (4.39) | 0.62 | 9.39 (3.82) | 9.26 (3.86) | | | 9.71 (3.69) | | | 0.25 | | | 13.74 (4.12) | 12.93 (4.21) | 14.78 (3.78) | **<0.01** |  |
|  | Men | 8.18 (3.89) | 10.73 (2.40) | 6.64 (3.83) | **<0.01** | 8.91 (4.04) | 9.24 (3.94) | | | 8.44 (4.17) | | | 0.29 | | | 9.10 (6.87) | 15.50 (4.87) | 6.87 (6.06) | **<0.01** |  |
|  | p | 0.06 | 0.44 | **<0.01** |  | 0.23 | 0.97 | | | 0.05 | | |  | | | **<0.001** | 0.10 | **<0.001** |  |  |
| Prior MH training | No | 9.32 (4.31) | 9.54 (4.15) | 9.15 (4.44) | 0.60 | 8.97 (3.75) | 8.90 (3.87) | | | 9.17 (3.41) | | | 0.49 | | | 10.17 (4.91) | 9.82 (4.36) | 10.77 (5.88) | 0.59 |  |
|  | Yes | 9.37 (4.38) | 10.89 (4.00) | 7.90 (4.28) | **<0.01** | 10.58 (4.07) | 11.07 (3.31) | | | 9.90 (4.88) | | | 0.12 | | | 13.62 (4.73) | 14.01 (3.84) | 13.24 (5.45) | 0.29 |  |
|  | p | 0.94 | 0.16 | 0.19 |  | **<0.001** | **<0.001** | | | 0.26 | | |  | | | **<0.001** | **<0.001** | 0.14 |  |  |
| M = mean; SD = standard deviation; n = absolute number; NA = not applicable; Prior MH training = prior mental health training; MHK = Mental Health Knowledge; pers DSS = personal Depression Stigma Subscale; openness= Openness to seeking help; Confidence= confidence in identifying or referring students; WS I = WhySchool I (2015-2016); WS II = WhySchool II (2018-2020); Human.= Humanities/Social Sciences. ^a^ cut-off point obtained from the decision trees. Statistical analysis included chi-square and fisher exact test for categorical variables, t-tests and ANOVA for continuous variables. | | | | | | | | | | | | | | | | | | | | |

Table S7 - Correlations of baseline outcomes scores (MHK, pers DSS, openness, and confidence) with each other and with training effects.

|  | **MHK at baseline** | **pers DSS at baseline** | **Openness at baseline** | **Confidence at baseline** |
| --- | --- | --- | --- | --- |
| MHK at baseline |  | -0.10^**^ | 0.12^**^ | **0.32^**^** |
| pers DSS at baseline | -0.01^*^ |  | -0.08^**^ | -0.05 |
| Openness at baseline | 0.12^**^ | -0.08^**^ |  | 0.12^**^ |
| Confidence at baseline | **0.32^**^** | -0.05 | 0.12^**^ |  |
| MHK gains, M(SD)= 24.10% (21.52) | **-0.79^**^** |  |  |  |
| pers DSS gains, M(SD)= -16.99% (16.23) |  | **-0.81**** |  |  |
| Openness gains, M(SD)= 9.30% (21.14) |  |  | **-0.59**** |  |
| Confidence gains, M(SD)= 17.93% (20.68) |  |  |  | **-0.59**** |
| MHK = Mental Health Knowledge; pers DSS = personal Depression Stigma Subscale; openness= Openness to seeking help; Confidence= confidence in identifying or referring students. Baseline scores correlations with each other are spearman’s rho, and with training effects are pearson; *p-value<0.05; **p-value <0.01 | | | | |

Table S8 - Final generalised linear models for each baseline outcome by profession and training level.

| **MHK** | | **B** | **SE** | **Wald χ²** | **p** | **95% CI** | |
| --- | --- | --- | --- | --- | --- | --- | --- |
| TL1 | Intercept | 7.50 | 0.17 | 1877.23 | <0.001 | 7.16 | 7.84 |
|  | Sex ^a^ | -0.73 | 0.20 | 13.52 | <0.001 | -1.12 | -0.34 |
| TL2 | Intercept | 7.21 | 0.083 | 7488.90 | <0.001 | 7.05 | 7.38 |
|  | Sex ^a^ | -0.32 | 0.12 | 9.23 | <0.01 | -0.53 | -0.11 |
|  | Prior training ^b^ | 0.28 | 0.10 | 7.69 | <0.01 | 0.08 | 0.48 |
| SHP | Intercept | 7.31 | 0.28 | 695.11 | <0.001 | 6.76 | 7.85 |
|  | Age 40-49 ^c^ | 0.45 | 0.22 | 4.07 | <0.05 | 0.01 | 0.88 |
|  | Prior training ^b^ | 0.85 | 0.27 | 10.57 | <0.01 | 0.34 | 1.37 |
| **pers DSS** | | **B** | **SE** | **Wald χ²** | **p** | **95% CI** | |
| TL1 | Intercept | 5.31 | 0.18 | 845.32 | <0.001 | 4.95 | 5.66 |
|  | WhySchool period ^d^ | 1.37 | 0.97 | 45.95 | <0.001 | 0.97 | 1.76 |
| TL2 | Intercept | 5.22 | 0.09 | 3381.69 | <0.001 | 5.38 | 5.39 |
|  | WhySchool period ^d^ | 1.50 | 0.11 | 182.86 | <0.001 | 1.72 | 1.72 |
| SHP | Intercept | 4.78 | 0.21 | 527.28 | <0.001 | 4.37 | 5.19 |
|  | WhySchool period ^d^ | 2.04 | 0.24 | 75.63 | <0.001 | 1.58 | 2.50 |
|  | Sex ^a^ | -0.54 | 0.23 | 5.56 | <0.05 | -0.98 | -0.90 |
| **Openness** |  | **B** | **SE** | **Wald χ²** | **p** | **95% CI** | |
| TL1 | Intercept | 9.23 | 0.16 | 3375.556 | <0.001 | 8.922 | 9.545 |
|  | WhySchool period ^d^ | -0.76 | 0.18 | 17.053 | <0.001 | -1.125 | -.401 |
|  | Sex ^a^ | -0.419 | 0.18 | 5.267 | <0.05 | -.777 | -.061 |
| TL2 | Intercept | 7.36 | 0.13 | 3039.12 | <0.001 | 7.10 | 7.62 |
|  | WhySchool period ^d^ | 1.22 | 0.17 | 53.73 | <0.001 | 0.89 | 1.55 |
|  | Region_Algarve ^e^ | 0.75 | 0.24 | 9.58 | <0.001 | 0.27 | 1.22 |
|  | Region_Lisbon ^e^ | 1.08 | 0.28 | 14.58 | <0.001 | 0.53 | 1.63 |
|  | Region_Alentejo ^e^ | 1.63 | 0.17 | 88.70 | <0.001 | 1.29 | 1.97 |
|  | Sex ^a^ | -0.65 | 0.16 | 16.67 | <0.001 | -0.97 | -0.34 |
| SHP | Intercept | 7.26 | 0.21 | 1204.19 | <0.001 | 6.84 | 7.66 |
|  | WhySchool period ^d^ | 1.51 | 0.24 | 39.60 | <0.001 | 1.04 | 1.98 |
|  | Region_Algarve ^e^ | 1.85 | 0.37 | 24.49 | <0.001 | 1.13 | 2.58 |
|  | Region_Lisbon+Setúbal ^e,f^ | 1.09 | 0.36 | 9.39 | <0.01 | 0.39 | 1.78 |
| **Confidence** |  | **B** | **SE** | **Wald χ²** | **p** | **95% CI** | |
| TL1 | Intercept | 1.82 | 0.27 | 44.89 | <0.001 | 1.29 | 2.36 |
|  | MHK | 0.01 | 0.001 | 15.49 | <0.001 | 0.01 | 0.02 |
|  | Age ≥ 50 ^c^ | 0.65 | 0.18 | 13.97 | <0.001 | 0.31 | 0.99 |
|  | Age 40 – 49 ^c^ | 0.49 | 0.18 | 7.70 | <0.01 | 0.14 | 0.84 |
| TL2 | Intercept | 2.36 | 0.10 | 533.12 | <0.001 | 2.16 | 2.56 |
|  | MHK | 0.01 | 0.001 | 50.31 | <0.001 | 0.01 | 0.02 |
|  | Prior training ^b^ | 0.22 | 0.06 | 13.05 | <0.001 | 0.10 | 0.16 |
| SHP | Intercept | 3.15 | 0.13 | 599.44 | <0.001 | 2.90 | 3.41 |
|  | Region_Algarve ^e^ | 0.42 | 0.18 | 5.62 | <0.05 | 0.07 | 0.77 |
|  | Region_Lisbon+Setúbal ^e,f^ | -0.36 | 0.171 | 4.46 | <0.05 | -0.70 | -0.03 |
|  | Age ≥ 50 ^c^ | 0.24 | 0.11 | 4.81 | <0.05 | 0.03 | 0.46 |
|  | Age 40 – 49 ^c^ | 0.33 | 0.10 | 10.75 | <0.01 | 0.13 | 0.53 |
|  | Sex ^a^ | -0.80 | 0.14 | 34.60 | <0.001 | -1.06 | -0.53 |
|  | Prior training ^b^ | 0.50 | 0.12 | 16.61 | <0.001 | -1.06 | -0.53 |
| MHK = Mental Health Knowledge; pers DSS = personal Depression Stigma Subscale; Opennes= openness to seeking help; Confidence= confidence in identifying or referring students; TL1 = teachers Level 1, TL2 = teachers Level 2, SHPs = school health professionals; B = beta regression coefficients; SE = standard errors; p =p-value; CI = confidence intervals. ^a^women, ^b^no, ^c^≤ 39 years old, ^d^WhySchool I (2015-2016), and ^e^North, are the reference group. All models are adjusted for WhySchool period and implementation region. ^f^ For level 1 teachers and SHPs groups, we merged the Centre region with the North and Setúbal with Lisbon to ensure statistical robustness, given the small number of participants in those areas. Probability distribution: normal, link function: power (0.5). | | | | | | | |
